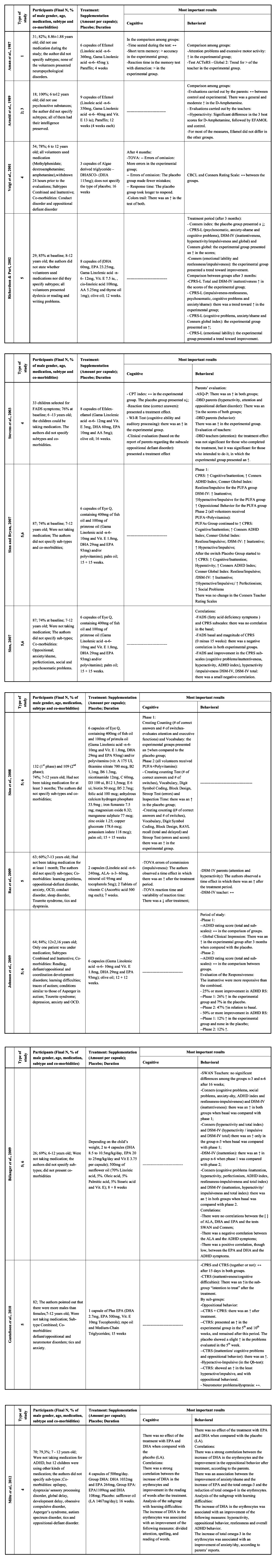# Effects of Low Doses of Polyunsaturated Fatty Acids on the Attention Deficit/Hyperactivity Disorder of Children: A Systematic Review 

**DOI:** 10.2174/1570159X11311020005

**Published:** 2013-03

**Authors:** Viviane Grassmann, Ruth Ferreira Santos-Galduróz, José Carlos Fernandes Galduróz

**Affiliations:** 1 Department of Psychobiology (Departamento de Psicobiologia) – Universidade Federal de São Paulo – Brazil; 2 Center of Mathematics, Computing and Cognition – Universidade Federal do ABC- Brazil; 3 Institute of Biosciences, Universidade Estadual Paulista, Laboratory of Physical Activity and Aging (LAFE)- Brazil

**Keywords:** Attention deficit/hyperactivity disorder; behavior, children, cognition, dietary supplements and polyunsaturated fatty acids.

## Abstract

Since attention deficit/hyperactivity disorder (ADHD) presents high prevalence among children, science has been researching alternative forms of treatment that do not involve medication. Objective: To evaluate the effects of polyunsaturated fatty acids (PUFAs) on attention deficit/hyperactivity disorder. Methods: We reviewed the articles published between 1980 and 2012 indexed in the databases PubMed, APA psychNET, Scopus and Web of Knowledge. Results: Initially 231 articles were selected, out of which 12 met the inclusion criteria. The articles selected reported a modest cognitive and behavioral improvement of the patients after treatment with low doses of PUFAs. Those results might be associated with the evaluation methodology, the doses of PUFAs administered or the duration of treatment.

## INTRODUCTION 

Attention deficit/hyperactivity disorder (ADHD) is a development alteration characterized by impulsiveness, excess of activity and limited capacity to keep attention [1,2]. Its prevalence all over the world is between 6.5% and 11% among children aged 5 to 15 years [3]. The neurobiological alterations include dysfunction in the dopaminergic transmission in the striatal structures and frontal lobes [4], and a lower brain volume [5]. These affected brain areas correspond to attention and executive functions (working memory, motor control and inhibition) [6]. 

The most common treatment modality involves the use of stimulants such as methylphenidate. They inhibit the reuptake of noradrenaline and dopamine by means of their transport protein, leading to an increase in the extracellular concentration of these catecholamines in the brain [7]. A study showed that 32% to 64% of the children still present significant levels of ADHD in spite of the treatment [8]. Therefore, it is important to develop new strategies to improve the treatment of this disorder. Some studies evaluated the effect of PUFAs such as omegas 3 and 6 on this disorder [9-18]. 

The two main families of PUFAs are omega 6 (linoleic acid) and omega 3 (alpha-linolenic acid). The metabolic product of linoleic acid is the arachidonic acid (AA), and those of the alpha-linolenic acid are the eicosapentaenoic (EPA) and the docosahexaenoic (DHA) acids [19]. These PUFAs might be utilized as mediators of immune and inflammatory responses and as energy source in several physiological systems and membrane structures [20]. These acids are essential for the central nervous system to work well, since about 50% of the brain weight consists of lipids, out of which 20% are PUFAs [21]. 

It is widely recognized that a diet deficient in omega 3 might influences neurotransmission, namely the dopaminergic and the serotonergic systems [22, 23]. As a result, the amount of dopamine can be reduced [24]. Conversely, supplementation with fish oil increases the level of dopamine and reduces monoamine oxidase B activities [25]. Therefore, as in the treatment with medication, the increase in the ingestion of omega 3 might enhance the central activity of dopamine in the pre-frontal cortex over time, thus reducing aggressiveness and impulsiveness [25-27]. 

Childhood is a critical and vulnerable period in which the supplementation with PUFAS is fundamental for the good working of the brain [28]. PUFAs, especially DHA and AA, accumulate rapidly in the gray matter of the brain [22] in this period, and its deficiency may cause deficits in memory, learning, mood and the sensorial system that can be irreversible [26]. Therefore, a diet rich in DHA might play a crucial role in the cognitive development and neural disorders of children [22]. 

Considering the relevance of this issue, we performed a systematic review of the literature in order to evaluate the effects of polyunsaturated fatty acids (PUFAs) on attention deficit/hyperactivity disorder. 

## METHOD 

We used the databases PubMed, APA psychNET, Scopus and Web of Knowledge to search for articles published between 1980 and 2012. We selected placebo-controlled studies in English that used supplementation with omega 3 and/or 6 to treat children with ADHD. The inclusion criteria were original and controlled articles that used PUFAs capsules supplementation, and evaluated the behavior and the cognitive functions of children with ADHD. In order to do so, we utilized the following boolean descriptors: (adhd OR attention deficit disorder OR attention deficit hyperactivity disorder OR attention-deficit/hyperactivity disorder) AND (omega OR docosahexaenoic acid OR alpha linolenic acid OR eicosapentaenoic acid OR arachidonic acid OR fish oil OR flax oil OR linseed oil OR polyunsaturated fatty acids). 

**Fig. (1):**
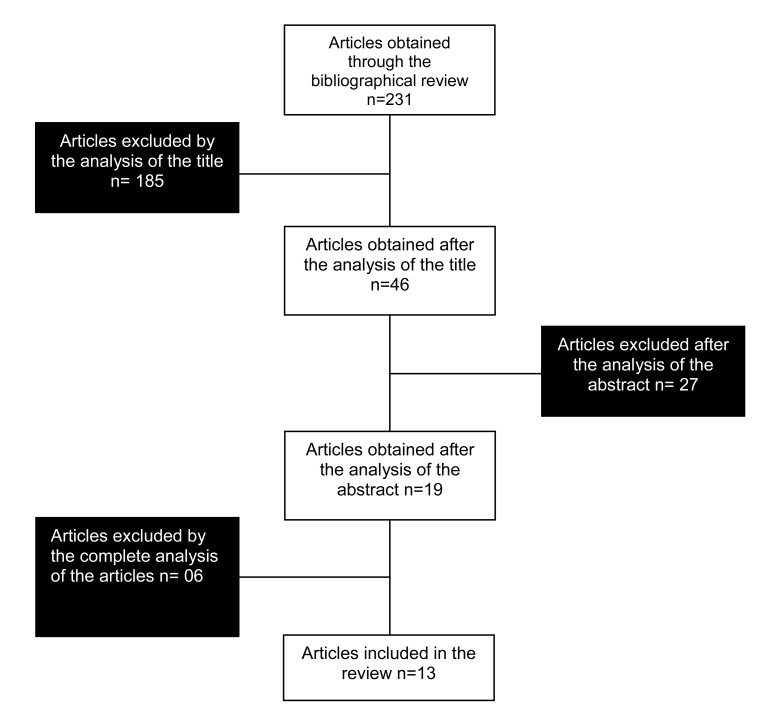
**Flowchart of the selection of studies**. After reading the title and the abstract we excluded the articles that did not meet the inclusion criteria. After reading the whole article we excluded two of them which did not used supplements, but only changed the diet of the participants; one which did not have a group with ADHD alone, and three which were not randomized.

We found 231 papers and selected only 13 of them (Fig.**1**) (Table **1**). Out of the 218 articles excluded, 79 were not original papers; 53 did not evaluate children with ADHD; 40 did not use supplementation with capsules of PUFAs; 23 evaluated neither children with ADHD nor supplementation; seven were carried out with adults; six studies evaluated animals; four did not assess the effect of supplementation on behavior and cognitive functions; three articles were not randomized; two were written in a language other than English, and one article was not found. 

## RESULTS 

### The Effect of PUFAs on Behavior 

The first study included 31 children of both genders who had ADHD and were not under medication, allocated to two groups (experimental and placebo). The experimental group ingested daily capsules containing 2,160 mg of linoleic acid and 270 mg of gamma linolenic acid (both omega 6) for four weeks, while the control group ingested liquid paraffin in the same period. The children improved in relation to attention and hyperactivity according to the reports of their parents and teachers [9]. 

However, Arnold *et al.* [10], in a similar study did not observe significant differences even when higher doses of omega 6 were used (2,800 mg of linoleic acid, 320 mg of gamma linolenic acid) and the treatment periods were longer (12 weeks). 

Two other articles [11, 17] evaluated the effect of omega 3 (EPA and DHA). The first study evaluated children who were already stable as a result of traditional medication (methylphenidate, dextroamphetamine or amphetamine) for ADHD. Nevertheless, their medication was withdrawn 24 hours prior to the evaluations. The volunteers were randomly allocated to two groups, placebo and experimental. The author did not state what kind of placebo was used. As for the experimental group, volunteers received capsules containing 345 mg of DHA (omega 3) for 16 weeks. This procedure did not improve the behavior of the children. However, it is important to emphasize that although the medication was discontinued prior to the evaluations, the T-score in the evaluation of the CBCL for parents indicated that the behavior of the children was already stabilized since the beginning, which can result in a ceiling effect [11]. 

The second study utilized both DHA and EPA (2.7 mg and 500 mg, respectively), and compared their effects with those of rape oil (placebo) for 15 weeks. After this period, Gustafsson *et al.* [17] observed improvement regarding inattention/cognitive problems, as reported by the teachers, only in the experimental group. 

Bélanger *et al.* [16] compared the effect of omega 3 (8,5 to 10,5 mg/kg/day of DHA and 20 to 25 mg/kg/day of EPA) with that of omega 6 (sunflower oil, which contains 70% of linoleic acid; 5% of oleic acid; 5% of palmitic acid, and 5% of stearic acid) for eight weeks. They observed improvement in hyperactivity and in the Conners global index only in the group that used omega 3. However, both groups showed improvement in cognition; anxiety-shy; social problems; restless-impulsive (Conners) and inattentive (DSM-IV). In the second phase of the same experiment, all the volunteers received omega 3. In this phase, those who had received omega 6 in the first phase presented similar results to those of the volunteers who received omega 3 in the first phase of the experiment. 

A more recent study (2012) compared the effect of a mixture of DHA (1,032 mg) and EPA (264 mg), and a mixture of EPA (1,109 mg) and DHA (108 mg) versus omega 6 (1,467 mg of linoleic acid- safflower oil). The authors selected children between seven and 12 years old diagnosed with ADHD, or who were in the 90^th^ or over percentile in the CPRS (Conners Parent Rating Scale). They did not observe differences between the treatment with EPA/DHA and placebo after four months of administration. However, there were correlations between the concentration of DHA in the erythrocytes and improvement in the oppositional behavior disorder according to parents’ reports, and also in learning difficulties [29]. 

Some studies evaluated the effects of a combination of omega 3 and omega 6. Richardson and Puri [12] treated children with ADHD-related problems with capsules containing 480 mg of DHA; 186 mg of EPA; 96 mg of gamma-linolenic acid; 864 mg of cis-linoleic acid, and 42 mg of AA, or olive oil as placebo for 12 weeks. The experimental group presented improvement in anxiety/shy tests; cognition (CPRS-L); inattentiveness; hyperactivity/impulsiveness; total DSM-IV index and Conners total global index. 

Stevens *et al.* [13] also administered a supplement that contained omega 3 and omega 6 (96 mg of gamma-linolenic acid; 480 mg of DHA; 80 mg of EPA, and 40 mg of AA) or placebo (olive oil) for 16 weeks to children who had fatty acids deficiency signs and symptoms (FADS) such as dry hair and skin, thirst and frequent diuresis. They detected improvement in the Anxiety Screening Questionnaire (ASQP) and Disruptive Behavior Disorders (DBD) questionnaires for parents in the subscales hyperactivity, attention and oppositional/defiant behavior in both groups. The experimental group, on the other hand, showed improvement only in the subscale conduct of the DBD, and in the subscale attention of the same instrument, according to their teachers’ evaluation. However, it is important to note that Sinn, [18] later showed that the FADS symptoms were related to linoleic acid deficiency, and both oils have it. 

In the study of Johnson *et al.* [14], the children received capsules containing 60 mg of gamma-linolenic acid; 174 mg of DHA and 558 mg of EPA for 12 weeks. Even though they presented improvement in the global clinical impression, their behavior after treatment was the same as that of the placebo group (olive oil). After evaluating responsiveness, they found that the inattentive were more responsive than the combined subtype, and found that among the children who improved 25% or more in the ADHD rating score, 26% were from the experimental group and only 7% were from the placebo group in the first phase, and 47% improved in the second phase in relation to baseline. However, among the children who improved 50% or more in the ADHD rating score, 12% of them were in the experimental group and none changed in the placebo group in the first phase, and 12% improved in the second phase when compared with baseline results. These authors also compared the subtypes of ADHD regarding the effect of PUFAs, observing that the children that underwent the treatment showed an improvement in the related to inattentive behavior. 

Raz *et al.* [15] detected no differences between the experimental (480 mg of linoleic acid and 120 mg of alpha-linolenic acid) and the placebo (vitamin C) groups. 

Sinn [18] evaluated children with both ADHD and FADS by selecting 87 children with ADHD and randomly assigning them to three groups. The PUFAs group received six daily capsules containing 60 mg of gamma linolenic acid, 174 mg of DHA and 558 mg of EPA; the PUFAs + Multivitamins received the same components mentioned above and vitamins; and the placebo group received palm oil. The author observed no correlation between FADS and CPRS subscales at baseline, but found a negative correlation between baseline FADS and the magnitude of CPRS, as well as a small correlation between FADS and the improvement in some sub-scales (Table **1**). 

In another paper [30], the same author showed an improvement in many behaviors (inattention, ADHD index, hyperactivity, impulsiveness and oppositional behavior) for the group that received PUFAs. In the second phase, when every child received PUFAs + Polyvitamins, the group that received only PUFAs in the first phase continued to improve their levels of attention, impulsiveness, hyperactivity and ADHD index. However, the group that had received placebo started to improve only in the second phase (attention, hyperactivity, ADHD index, impulsiveness, perfectionism and social problems). 

### Effects of Polyunsaturated Fatty Acids on Cognitive Functions 

Aman *et al.* [9] evaluated motor skills, attention and short term memory. Their results showed greater accuracy in the latter after treatment with omega 6. On the other hand, Voigt *et al.* [11] utilized DHA (omega 3) as supplement and detected no differences between the placebo and the experimental groups. Milte *et al.* [29], in a similar study, found differences between the placebo and the experimental groups only in the concentrations of DHA in erythrocytes and in the reading of words after treatment. 

Stevens *et al.* [13] used both omega 3 and omega 6 as supplement, detecting no significant differences in the Continuous Performance Test (CPT) index of the expermental group. In another study, Sinn *et al.* [31] focused on the cognition of ADHD patients and observed improvement in their level of attention and executive functions evaluated by the Creature Counting test after 15 weeks. Finally, Raz *et al.* [15] administered PUFAs + Multivitamins for seven weeks and observed no differences between the groups. 

## DISCUSSION 

The present review indicated a possible improvement in the symptomatology of the ADHD, but more specific studies are necessary with methodological accuracy and the exclusion of comorbidities. 

The major problem we found was a discrepancy in the doses of PUFAs used in the different researches. This finding might reflect the need of further studies that establish a therapeutic dose. Some studies suggest the ratio of 4:1 between omega 6 and 3 for an ideal diet [28]. In spite of that shortcoming, we could notice that the studies which used a combination of omega 3 and omega 6 presented better results. 

On the other hand, it is possible that the doses used in these studies were too low to show better results. Other studies, which did not use the placebo, showed that high doses of EPA and DHA have evident therapeutic benefits [32,33]. Germano *et al*. [32] used 0,234 g/day/kg of fish oil, which has at least 75% of omega 3, in children with a mean weight of 33,97 kg for eight weeks. Sorgi *et al*. [33], used 16.2 g EPA/DHA (10.8g EPA and 5.4g DHA) concentrates per day for four week and then decreased this dose based on the AA:EPA ratio in the isolated plasma phospholipids. The children that were below 1.0 on AA:EPA had the ratio decreased to the dose of 8.1 g (2.7 g DHA, 5.4 g EPA per day), and the children between 1.0 and 1.5 AA:EPA ratio had the dosage decreased to 12,1 g (4 g DHA, 8.1 g EPA per day) for the following four weeks. Both studies decreased the AA/EPA ratio after 8 weeks, and this reduction resulted in an improvement in behavior (inattention and hyper-activity). 

Another shortcoming is the lack of standardization regarding the evaluation and the time of treatment, since there was great variation (between four and 16 weeks) even among studies that yielded positive results. 

In this sense, Raz *et al*., [15] used a small amount of the short chain omega-3 (rather than EPA/DHA) for seven weeks and did not find a difference between the placebo and the experimental group. Both groups showed improvement, which can mean a placebo effect. 

Independently of the time and dose, five studies [9, 11, 13, 16, 17, 29] showed incorporation of the supplement, as evaluated through blood tests. However, the other studies did not observe this incorporation, so it is difficult to establish whether the patients real consumption the PUFAs. 

Even though the evaluation of omega intake is important, only three out of the 12 studies analyzed carried out such control [13, 16, 17]. 

It is a known fact that ADHD is subdivided into three subtypes: inattentive; hyperactive-impulsive, and combined hyperactive-impulsive + inattentive [1]. However, only three studies reported the subtypes of their samples. Voigt *et al.* [11] and Johnson *et al.* [14] studied children of the subtypes inattentive and combined, while Gustafsson *et al.* [17] evaluated only those who presented the combined subtype. Only the study of Johnson *et al. *[14] compared the effect of PUFAs among the subtypes, they observed a greater improvement in the behavior of the inattentive individuals who underwent the treatment. This lack of comparison in the other studies makes it difficult to establish whether this treatment would be beneficial for children who are more inattentive, hyperactive or present a combination of both subtypes. PUFAs seem to yield better results in the improvement of hyperactivity and inattentiveness. However, if we separate individuals by subtypes, it is possible that each group has a different benefit. 

Another aspect regards the co-morbidities. Children with ADHD usually present associated psychiatric conditions, among which we can mention mood disorders; oppositional defiant disorder; conduct disorder; tics, and Tourette syndrome as the most common [34]. Only one study reported no co-morbidities in their volunteers [16]. The others either omitted that kind of information or reported different co-morbidities. However, they did not compare their effects, except for two studies which evaluated the effect on ADHD as well as on its associated co-morbidities. The first one [17], showed greater benefits of supplementation with omega in children who presented oppositional defiant disorder, showing that part of the positive outcomes of the treatment with PUFAs might be due not only to the improvement of ADHD, but rather to a global improvement that includes the co-morbidities. The other study [29] showed that there is a relation between n-3 and the literacy/behavior in ADHD children. When they analyzed the subgroup with learning difficulties, they found that the increase of DHA in the erythrocytes was associated with an improvement in some measures (hyperactivity, oppositional behavior, restlessness and overall ADHD behavior). Furthermore, the children with learning difficulties had lower DHA and total n-3 PUFA and higher AA/EPA ratio than those without. In this sense, it would be interesting for researchers to carry out studies that evaluate children who do not present co-morbidities, comparing them to those who do. 

Although the randomized and controlled study of Richardson & Montgomery [35] is not part of this systematic review, since the primary aim of that study was to analyze the effects of PUFAs on children with developmental coordination disorder, they found an improvement in reading and spelling and in many behaviors that are related to ADHD in those children (opposition, cognitive problems, hyperactivity, anxiety, social problems, impulsivity, emotional lability, inattention and ADHD indexes). 

It is important to stress the fact that few studies used really inert substances as placebo. Some authors used other oils (olive, rape, safflower and palm) or vitamin C for that purpose. Even though it is not known whether vitamin C or those oils used as placebo have a beneficial effect on children with ADHD, some authors used those components, which could result in a global improvement of those individuals. Milte *et al.* [29] detected several correlations between DHA and EPA in the erythrocytes versus behavior and/or cognitive functions, but did not observe the effect of treatment. Probably they did not observe treatment effects because of the placebo they used, since this component (rich in linoleic acid – n-6) might have masked the effect of treatment. 

The low to moderate improvement showed in the behavior of the experimental groups in three studies [12-14] might have been more pronounced had the authors used another type of placebo, since olive oil also contains omegas 3 and 6, even if it is in small amounts. Likewise, Stevens *et al.* [13] reported a cognitive improvement in the experimental group that could have been more outstanding, since olive oil also seems to have the property to enhance cognition. Even though it is a monounsaturated fat, it contains antioxidant properties [36]. Another important factor is that olive oil contains oleic acid (omega 9), which is converted into ethanolamine in the intestines. This substance seems to improve the consolidation of memory in animals through an autonomic signaling that results in a noradrenergic activation in the amygdala [37]. 

The placebo used in the study of Gustafsson *et al.* [17] also contains oleic acid. Those authors used rape oil which, according to Hamid *et al. *[38], contains 15.6 to 24.3% of oleic acid, in addition to 12 to 13.7% of linoleic acid, 1.9 to 2.1% of linolenic acid and 2.0 to 2.9% of arachidonic acid, and therefore might have influenced the results. Similarly, vitamin C (used in the study of Raz *et al.* [15]) is an active compound which has antioxidant properties that can influence cognitive performance and behavior [39]. Palm oil, on the other hand, is rich in vitamin E. Alpha-tocotrienol, one of its derivatives, is a neuroprotective agent in the arachidonic acid cascade that can act on the enzymatic and non-enzymatic mechanisms in a brain lesion [40], but it is important to point that the active oil had vitamin E as well. 

In addition to the diversity of placebos, we should also stress that the treatment with stimulants was not interrupted [11, 13, 14], which could have masked the effect of the treatment with PUFAs, since the stimulant *per se* causes a beneficial effect on those patients. In the experiment of Stevens *et al*. [13], more than half of the patients were taking medication for ADHD, but Arnold *et al.* [10] observed that omega 6 has an intermediary effect between placebo and D-amphetamine. Moreover, the volunteers in the study of Voigt *et al.* [11] could have been undergoing treatment with medication, but they were supposed to discontinue its use 24 hours prior to the evaluation. This could result in an experimental bias, since caregivers of those children might have misinterpreted the effects, that is, they would not know for sure whether the results were due to the PUFAs or to the withdrawal of medication.

Another important aspect to point out is the sample size. Since some children dropped out before the end of many studies that are part of this review, they had a small sample size, which can influence the statistic power of the evaluations. . 

Finally, it seems that the relation between the ingestion of capsules containing PUFAs and ADHD has not been totally established [41], since other studies performed to date are very heterogeneous, which makes their interpretation difficult [23]. However, it is possible that higher doses of PUFAs would provide significant benefits, and therefore future researches could do a randomized and controlled study with higher amounts of PUFAs for the treatment of ADHD. 

## DISCLOSURE OF FUNDING 

This article received funding for research from FAPESP (# 2011/ 08387-6), *AFIP, FAPESP, and CNPq*. 

## Figures and Tables

**Table 1:** Review of the Articles Analyzed in this Systematic Review